# The Promise and Pitfalls of Artificial Intelligence in the Evaluation of Synthetic “Greenness”

**DOI:** 10.1002/cssc.202500809

**Published:** 2025-08-21

**Authors:** Paweł Mateusz Nowak, Michał Woźniakiewicz, Grzegorz J. Nalepa, Bartosz A. Grzybowski

**Affiliations:** ^1^ Laboratory for Forensic Chemistry Department of Analytical Chemistry Faculty of Chemistry Jagiellonian University in Kraków ul. Gronostajowa 2 30‐387 Kraków Poland; ^2^ Jagiellonian Human‐Centered AI Lab Mark Kac Center for Complex Systems Research Institute of Applied Computer Science Faculty of Physics Astronomy and Applied Computer Science Jagiellonian University ul. prof. Stanisława Łojasiewicza 11 30‐348 Krakow Poland; ^3^ IBS Center for Algorithmic and Robotized Synthesis (CARS) and the Department of Chemistry Ulsan National Institute of Science and Technology Ulsan 44919 Republic of Korea

**Keywords:** artificial intelligence, green chemistry, greenness metrics, machine learning, synthesis planning algorithms

## Abstract

Almost three decades after the formulation of Anastas’ 12 guiding principles, there is still no consensus on how to best quantify the greenness of synthetic routes—instead, heterogeneous metrics abound that vary in assumptions and scope. This perspective argues that since greenness is an inherently multiparametric concept, its quantification can be aided by modern artificial intelligence (AI), methods that have already proven extremely powerful in establishing correlations “hidden” in large, multivariate data. Given, however, that even the cutting‐edge AI tools cannot yet evaluate the greenness of synthetic procedures without extensive prompting, alternative approaches are also considered, in which greenness‐oriented AI is trained under the guidance of human experts and using appropriately selected corpus of green versus nongreen synthetic examples. Furthermore, it is suggested that models emerging from any such studies will make most impact if incorporated into the rapidly developing, AI‐driven synthesis design algorithms. These algorithms are now gaining wider community acceptance and may soon guide which syntheses are prioritized for experimental execution. It is important that greenness metrics affirm themselves as part of this prioritization, making gradual but steady impact on the greenness of synthetic chemistry at large.

## Graphical abstract

Rapid rise of AI prompts a question whether it can be used to accelerate and standardize assessment of synthetic “greenness.” This perspective argues for the potential of such applications, although it also identifies several hurdles, like the need for extensive training or the creation of “ground truth” datasets.

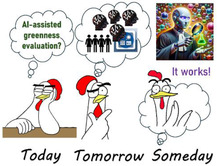

## 1 Introduction

The pursuit of sustainable development goals is reshaping many aspects of chemistry and related sciences.^[^ ^1^, ^2^, ^3^, ^4^ ^]^ Green chemistry (GC) and green analytical chemistry (GAC)—a subfield focusing on analytical methods—are the cornerstones of this global effort and are garnering widespread interest.^[^ ^5^, ^6^, ^7^, ^8^ ^]^ Nonetheless, despite an increasing base of supporters and influx of funds from both governmental and private sources, GC's further development is not without challenges. Although almost three decades have passed since Anastas and Warner had formulated their 12 guiding principles of GC,^[^ ^3^ ^]^ ambiguities still arise when assessing greenness in a comprehensive and objective manner. The need to standardize greenness as an attribute characterizing specific synthetic methods or processes was discussed by Curzons et al. already in 2001,^[^ ^9^ ^]^ but still remains an open challenge.^[^ ^10^, ^11^, ^12^ ^]^ This is concerning as terms such as “green,” “sustainable,” and “eco” are often being misused for the purpose of branding and monetary gain, which damages the reputation of GC as a scientific discipline. Therefore, there is some urgency in providing reliable means of assessing greenness via more transparent metrics and based on more objective standards.

**Figure 1:**
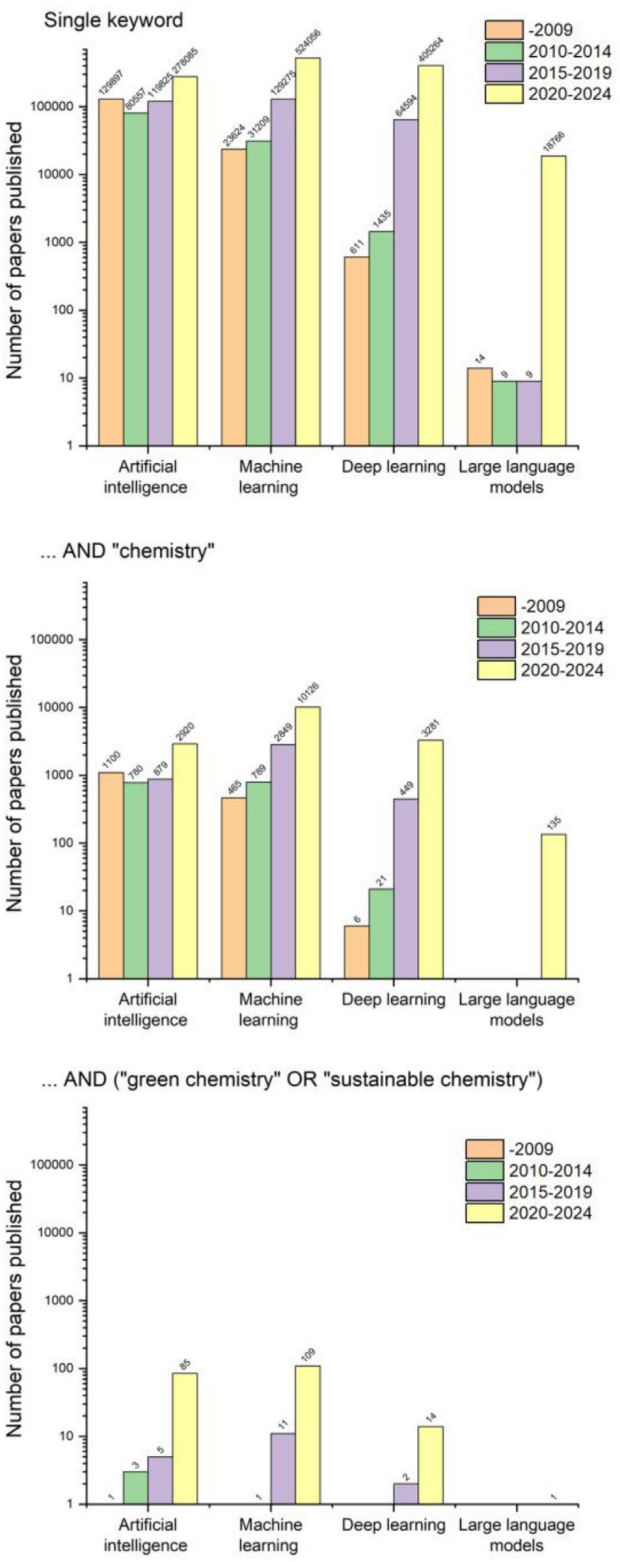
The number of published papers (on a logarithmic scale) found in the Scopus database (October 2024) for which the terms “artificial intelligence,” “machine learning,” “deep learning,” or “large language models” were present in the title, abstract, or keywords—upper chart; was additionally accompanied by “chemistry” term—middle chart; or by more specific “green chemistry” or “sustainable chemistry” terms—lower chart. Four different publication periods were considered.

One of the questions this perspective aims to pose is whether such standardization of synthetic greenness can be fostered by artificial intelligence (AI) and machine learning (ML). These techniques have proven extremely powerful in identifying “hidden” correlations in multivariate data and, as such, may lend themselves to the multiparametric evaluation of synthetic greenness. In this context, we emphasize a growing interest in AI/ML in the GC community (bottom histogram in **Figure** **1**). Yet, we also note a rather scattered effort ranging from ecology to nanosynthesis, to catalyst design, to process optimization. These applications, briefly summarized in Section 2, are all valuable but they also carry some risk of proliferating individual AI/ML metrics and models that are not generalizable beyond the specific processes and training datasets. Instead, what motivates our discussion here is whether AI/ML can play a more unifying role that is applicable to all kinds of synthetic processes. Can AI learn about the metrics of greenness by “reading” the relevant literature categorized by the experts into “green” and “not green”? Can it then apply them to evaluate synthetic procedures? Can it identify greenness metrics that offer some level of objectivity? As we will see in Section 3.2, the answers to these questions are nuanced. On one hand, state‐of‐the‐art AI can very rapidly absorb synthetic knowledge and greenness metrics to evaluate synthetic protocols—on the other hand, these evaluations are not always correct and require human supervision. This points toward other schemes in which AI is supervised or trained by human experts. It also makes us consider, in Sections 3.3, 3.4, 3.5, how such hybrid, human–AI efforts could be structured (and funded), and which methods and datasets could be used.

Assuming that some AI‐driven methods for evaluating synthetic greenness become, in due time, available, we then ponder how they should be popularized to make a real‐world impact (Section 4). One can certainly envision a top‐down approach in which some governing body or funding agency mandates its grant recipients to use a particular algorithm to evaluate synthetic routes they wish to publish. However, such top‐down policies are not always effective (there are already a myriad of greenness and sustainability directives having limited practical impact). Instead, we suggest a “bottom‐up” alternative—based on AI itself—whereby metrics of greenness are incorporated into the scoring functions of the rapidly developing synthesis‐planning programs. These programs typically offer tens or even hundreds of viable synthetic routes and “greenness” may be one consideration that ultimately biases the choice of routes committed to experiment. As the technology is being adopted and commercialized (which is happening now), the number of green syntheses designed by machines will increase, making gradual impact on synthesis at large.

Naturally, this process will take time but it will happen. Twenty–thirty years ago, chemists designed syntheses by paper‐and‐pencil, with requisite visits to the library; nowadays, few go to any library and the design process is already semiautomatic, supplementing human thinking with searches of databases such as Reaxys or SciFinder; in 10 years it will be predominantly automatic, with complete routes designed autonomously by computers. We feel it is an immense opportunity for GC to act now to have its principles incorporated into these synthetic planners of the future.

## 2 Current Applications AI in Synthesis‐Oriented GC

With this vision in mind, let us begin by briefly outlining a broad spectrum of problems related to green synthesis already studied with the help of AI/ML (**Table** **1**). This is, by far, not a comprehensive review and does not encompass interesting—though less relevant to this perspective—studies focusing on, e.g., environmental ecology^[^ ^13^ ^]^ or sustainable energy.^[^ ^14^ ^]^

**Table 1:** Selected applications of AI‐based tools in synthesis‐related GC.

Aim of using AI in the study	AI methods	Results
Use of Chematica/Synthia software to design syntheses of biologically active substances that have proven synthetically challenging to human experts^[^ ^19^, ^23^, ^24^ ^]^	Multipriority queue search of synthetic space using anthropomorphic scoring functions	Experimental syntheses of drugs offering improved yields and atom economy. First in class, AI‐designed and experiment‐validated syntheses of complex natural products, including examples of dramatic reduction is synthetic length compared to prior literature
The use of Allchemy software for the green syntheses commencing from industrial wastes and leading to numerous essential drugs^[^ ^30^ ^]^	Forward reaction networks guided by process‐aware scoring functions	Experimentally validated laboratory‐scale syntheses as well as flow‐chemical production schemes on ≈kg scales and including the metrics of process greenness
Augmentation of synthesis planning with green solvent recommendations^[^ ^25^ ^]^	Monte Carlo Tree Search (MCTS) trained with reinforcement learning	Identification of shorter and potentially greener synthesis routes
Optimization of a synthesis process for fluorescent carbon dots (CDs) derived from tapioca, in line with green chemistry principles for sustainable synthesis^[^ ^15^ ^]^	ANN‐MLP with the fast LM	Synthesis of fluorescent CDs were in excellent agreement with ANN predictions. The process was optimized to reduce or eliminate carbon footprint
Size optimization of cobalt oxide nanoparticles synthesized using a plant‐mediated method^[^ ^16^ ^]^	ANN‐MLP‐LM	ANN model provided accurate estimates of biosynthetically prepared cobalt nanocrystals (CoNCs)
Green synthesis of nanomaterials for selective removal of cationic dyes^[^ ^17^ ^]^	ANN	Predictions of the ANN model agreed with experimental data
Application of ML to quantitative structure–property relationship (QSPR) to identify key structural elements and crystallinity of covalent organic frameworks (COFs) synthesized using green solvents^[^ ^80^ ^]^	kNN, SVM, decision tree, RF, ANN, adaptive boosting, naïve Bayes, and quadratic classifiers	ML‐based methodology offered high‐quality predictions of COFs’ crystallinity and surface area. Experiments confirmed the robustness of the model and its applicability to new COFs
Development of green spectrophotometric methods for the determination of coenzyme Q10 and vitamin E in synthetic mixtures and in a pharmaceutical preparations without a preseparation step^[^ ^81^ ^]^	ANN	ANN‐based spectrophotometric determination method proved efficient in determining both active ingredients over extended nonlinear ranges (nonideal conditions). ANN combined advantages of high accuracy and specificity with the speed of the spectrophotometric method
Toxicity analysis of ternary mixtures with data derived from high‐throughput, ink‐jet printing experiments^[^ ^82^ ^]^	Linear regression (LR), kNN, SVM, adaptive boosting (AdaBoost), gradient boosted regression trees (GBRT), RF	Concentrations of thousands of ternary‐mixtures could be used as multidimensional input to a ML model to predict their acute toxic effects. RF algorithm showed highest learning efficiency and prediction accuracy

ANN, artificial neural networks; ANN‐MLP, ANN‐based prediction model with multilayer perception; RF, random forest; GNN, graph neural network; kNN, *k*‐nearest neighbors; LM, Levenberg–Marquardt backpropagation algorithm; ML, machine learning; RSM, response surface methodology; SVM, support vector machine.

### 2.1 Synthesis of Functional Nanomaterials

In several works centered on nanomaterials, ML aided the design of green syntheses of quantum dots^[^ ^15^ ^]^ and other nanocrystals,^[^ ^16^ ^]^ including those to be used in remediation processes.^[^ ^17^ ^]^

### 2.2 Organic Synthesis

With computer‐aided organic synthesis under rapid development,^[^ ^18^, ^19^, ^20^, ^21^, ^22^, ^23^, ^24^ ^]^ some works have already considered the greenness aspects of the proposed routes. For instance, Jensen and co‐workers^[^ ^25^ ^]^ have incorporated information about green solvents into their algorithms. In a related effort, Bystrzanowska and Tobiszewski used various ML techniques to recommend sustainable solvents, reagents, processes, or conditions of processes.^[^ ^26^ ^]^ Furthermore, Tobiszewski et al. developed a solvent selection system based on a combination of chemometrics and TOPSIS multicriteria decision analysis.^[^ ^27^ ^]^ Sels et al. trained a Sustainable Solvents Selection and Substitution Software (SUSSOL)^[^ ^28^ ^]^ on a set of ≈500 solvents, of which 118 have been characterized as “green” based on the CHEM21 methodology.^[^ ^29^ ^]^ This database can become a valuable starting point to test green solvent alternatives in industrial processes. In turn, we used more extensive—yet still heuristic—scoring functions incentivizing retrosynthetic searches not only to choose greener solvents but also to avoid toxic intermediates and by‐products, eliminate steps requiring energy‐intensive methods, and prioritize routes characterized by the lowest possible cumulative process‐mass‐intensity (cPMI indices). Such process‐aware scoring functions were used to design synthetic methods that convert industrial chemical wastes into essential medicines.^[^ ^30^ ^]^ In a more recent work,^[^ ^31^ ^]^ we used computer‐planning to revalorize larger waste molecules into smaller but value‐added ones. Several computer‐designed plans from these works were subsequently validated by experiment on both laboratory and pilot scales, the latter using synthesis automation developed by the On‐Demand Pharmaceuticals company.

### 2.3 Life Cycle Assessment

In an interesting extension beyond synthesis design, Zhu et al.^[^ ^32^ ^]^ applied ML to seek greener chemicals for already existing industrial processes. Focusing on the synthesis of antidiabetic sitagliptin, they identified trifluoroacetic anhydride as the most environmentally problematic component. A scheme based on massive similarity screening followed by deep neural network analysis was then used to identify alternative chemicals to improve the process in terms of life cycle assessment.^[^ ^33^ ^]^ As many as 17 such chemicals were identified with 1,2‐ethanediyl ester suggested as the optimal replacement.

### 2.4 Catalyst Design

Chemical AI is also contributing to the discovery and optimization of catalysts whose use is advocated under Anastas’ 12 guiding principles. Recently, major experimental breakthroughs have been achieved by the groups of Sigman,^[^ ^34^, ^35^ ^]^ and Denmark^[^ ^36^, ^37^ ^]^ (for mononuclear catalysts) as well as Schoenbeck,^[^ ^38^ ^]^ who demonstrated ML design of dinuclear catalysts. In turn, Grzybowski and Mlynarski groups^[^ ^39^ ^]^ used ML to suggest catalyst replacements most effective for a given reaction of interest. All of these models have been accompanied by experimental validations, including examples of large‐scale, industrial applications of AI‐designed catalysts.^[^ ^40^ ^]^

### 2.5 Optimization of Reaction Conditions

Synthesis design is closely related to reaction optimization in multidimensional spaces of conditions, preferably taking into account monetary cost, ease of execution, energy consumption, atom economy, production of waste, and toxicity of reagents/disposables. Some of the notable advances in the AI‐driven optimization arena include the landmark works of Doyle and co‐workers on the Bayesian optimization of individual reactions,^[^ ^41^ ^]^ and Grzybowski and Burke on the optimization of conditions across a wide spectrum of substrates to ensure maximal generality of a given reaction methodology,^[^ ^42^ ^]^ and thus to limit the number of different reagents/catalysts used for different substrates. Such optimization campaigns may and should extend beyond laboratory‐scale reactions. In one industrially relevant example, Kim et al. used AI to optimize parameters of nonoxidative methane conversion.^[^ ^43^ ^]^ In all of these works, AI's predictions were accompanied by experiments. In ref. [42], AI optimizer achieved a very notable result of outperforming the multiple‐year‐long efforts of human experts on the very same problem, increasing the average yield (compared to the human state‐of‐the‐art) by roughly twice.

### 2.6 Discovery of New, Efficient Reactions

While the optimization efforts focus on the already known types of reactions and processes, AI can also assist in the exploration of novel chemical structures. In the context of GC, the combination of AI and laboratory automation can prove a powerful approach. For instance, Cronin's group^[^ ^44^ ^]^ demonstrated that it can streamline reaction discovery while minimizing spurious and, therefore, wasteful experimentation. In turn, we approached this problem from a theory‐driven angle, whereby AI was used to design and propagate networks of mechanistic steps from which mutually compatible and efficient sequences were then chosen.^[^ ^45^ ^]^ This effort led to the discovery of >10 mechanistically unprecedented reactions, including multicomponent, MCR, which are sought in GC because they can build complex scaffolds in one step, with high atom economy and without any purification of intermediates.

## 3 AI for the Quantification of Synthetic Greenness

### 3.1 Current Status

However valuable, the diverse examples discussed in the previous section do not address what we believe is one of the major shortcomings of GC—namely, that greenness is often in the eye of the beholder rather than based on universally approved criteria. In fact, numerous tools and metrics for assessing greenness have been proposed, though their sheer number prevents detailed scrutiny here, especially given that excellent reviews on the topic are available.^[^ ^46^, ^47^, ^48^ ^]^ For the sake of our discussion, it is important to note the heterogeneity of these metrics: some are simple indicators providing numerical values for further analysis and evaluation (e.g., E‐factor^[^ ^49^ ^]^), whereas others are more complex models operating according to some preadopted patterns, e.g., Eco‐Scale,^[^ ^50^ ^]^ Analytical Eco‐Scale,^[^ ^51^ ^]^ or other models used in GAC.^[^ ^10^, ^11^, ^52^, ^53^ ^]^ They also differ in structure, in the way of expressing and visualizing the results, and in the criteria selected for assessment. Our own recent attempts to address some of these challenges include the concepts of “Unified Greenness Theory” providing a theoretical description of selected aspects of greenness^[^ ^10^ ^]^ and of “white chemistry” aimed at reconciling greenness with functionality.^[^ ^10^, ^11^, ^12^ ^]^ It should be noted, however, these efforts do not yet assure full objectivity or even widespread consensus.

On one hand, such a diversity of tools may be beneficial, as they can complement one another. On the other hand, the information these tools aim to capture may be inconsistent and scattered, making it difficult for holistic interpretation and complicating the standardization of greenness. Moreover, the use of greenness metrics often requires specialized knowledge, laboratory experience, additional effort and time—in particular when the model application is laborious or if some data needs to be extracted from extensive literature. Thus, there is also an inherent human bias as scientists tend to use tools that are simple and quick to use, although these benefits may come at the expense of comprehensiveness. Faced with such challenges, we wondered whether state‐of‐the‐art AI methods could be of help, either to automate greenness evaluation of chemical procedures using known or new metrics or to derive universal greenness standards that would unify assessment results obtained with different existing metrics.

### 3.2 Testing Large Language Models for Automated Greenness Evaluation

For an AI method to automatically evaluate greenness of a given chemical procedure, it should be able to first “read” and “understand” its key points and then quantify them according to some GC metric. The ability to read and understand brings to mind the famed large language models (LLMs). Wondering how such algorithms would perform in realistic synthetic problems, we performed an experiment, in which Open AI's ChatGPT 4.0 was further trained for greenness assessment using the so‐called ChlorTox Scale—a greenness metric designed to evaluate risks associated with chemical reagents.^[^ ^54^, ^55^ ^]^

Briefly, the key assumption of ChlorTox Scale is to express—akin to toxic equivalency factors familiar from environmental toxicology^[^ ^56^, ^57^ ^]^—the hazards of different substances relative to the hazard of a common standard, the thoroughly safety‐ and toxicity‐tested chloroform. For any substance of interest, the risk value is calculated as a function of various hazards (poisoning, chemical burns, irritation, carcinogenicity) retrieved from the safety datasheets and presented in the so‐called Globally Harmonized System of the Classification and Labelling of Chemicals format. Comparison of hazards between the substance of interest and chloroform provides its relative hazard measure, while the exposure to hazard is proportional to its mass

(1) $$\text{ChlorTox}=\frac{{\text{CH}}_{\text{sub}}}{{\text{CH}}_{{\text{CHCl}}_{3}}}\times m_{\text{sub}}$$

where the ChlorTox value, expressed in the mass of chloroform [g], reflects a degree of chemical risk associated with the substance‐of‐interest, considering its properties (hazards) and the amount used. The ${\text{CH}}_{\text{sub}}{\text{CH}}_{{\text{CHCl}}_{3}}$ term represents a relative chemical hazard (hazard of using the assessed substance in relation to chloroform, assuming the same mass‐to‐volume concentration of both chemicals), and *m*_sub_ is a mass of the substance‐of‐interest to be used in a particular procedure. The ChlorTox values characterizing different substances can be added to express the total chemical risk predicted for the entire procedure (Total ChlorTox). The ChlorTox Scale is suitable for assessing both analytical and synthetic methods. In the latter case, the mass of the individual reagents should be counted, for example, per 1 g of the final product.

Our ChatGPT experiment, detailed in the Supporting Information, was divided into three phases. In Phase I, we tested ChatGPT's current knowledge on topics related to GC and greenness metrics and also improved our communication skills with ChatGPT in terms of prompt writing, acceptable file formats, avoidance of issues related to interpretation of numerical data, etc.^[^ ^58^ ^]^ We observed that the chatbot already had rudimentary textbook knowledge of the main assumptions of GC, but not of the specific assessment tools, including the ChlorTox Scale (which was published only in 2023,^[^ ^59^ ^]^ and, perhaps, not yet updated in ChatGPT's knowledge base).

In Phase II, we began training our own chatbot (ChlorTox Analyst). As primary input, we shared the texts of key publications describing the ChlorTox Scale^[^ ^54^ ^]^ and the related database (ChlorTox Base),^[^ ^55^ ^]^ which contained data on chemical hazards of ≈700 reagents (${\text{CH}}_{\text{sub}}{\text{CH}}_{{\text{CHCl}}_{3}}$ in Equation (1) was determined based on the Weighted Hazards Number,^[^ ^54^, ^55^ ^]^ WHN, calculated based on the safety data sheets). As a training set, we used examples from an organic chemistry course book providing detailed protocols for the synthesis of simple small molecules.^[^ ^60^ ^]^ In doing so, we focused on five entities: 2,3‐dibromo‐3‐phenylpropanoic acid, 4‐methylacetanilide, cyclohexyl acetate, acetanilide, and acetylsalicylic acid. If there were no hazard data in the database, the ChlorTox Analyst was asked to independently calculate the WHN values based on the safety data sheets provided by us as additional input. In each case, ChlorTox Analyst was ultimately able to calculate correctly the Total ChlorTox values—however, it made numerous mistakes along the way. The most common error was to assume incorrect WHN values needed for assessing chemical hazards. This, in turn, was due to reading incorrect numerical values from an Excel file of the ChlorTox Base (eventually solved by using a pdf file exported from xlsx), or due to making some unjustified approximations. For instance, in the absence of data for a given reagent, the AI used data for other substances. It also often assumed that the amounts of certain substances were negligible (even though this was not true), or that certain substances do not pose any hazard (which was also incorrect). These assumptions were in no way communicated by the chatbot to the user and required meticulous postanalysis of why the output differed from the expected ground truth. The records of all conversations (see Supporting Information) evidence that the number of prompts leading to the final, correct outcome was quite significant, between five and eight per synthesis.

**Figure 2:**
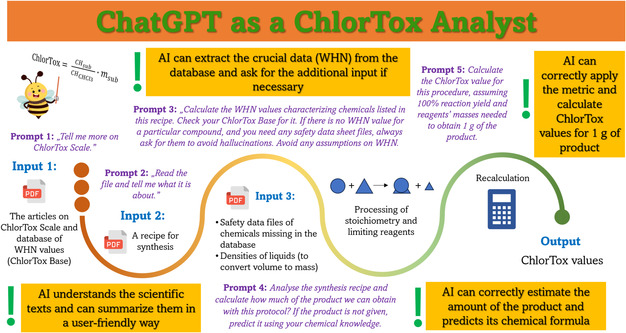
Flowchart of using ChlorTox Analyst assisting in the evaluation of synthetic procedures using the ChlorTox Scale (one of the available greenness metrics), including prompts to be used in a dialogue with a chatbot, types of input files, and identified chatbot's competences. This flowchart is suggested based on our own experiences but does not guarantee the avoidance of LLM's errors and hallucinations, necessitating expert control and verification of the output.

Based on these outcomes, we optimized the wording of the prompts (see **Figure** **2**), which we hoped would reduce the aforementioned mistakes. Specifically, in Phase III, we used ChlorTox Analyst with the knowledge gained in Phase II, communicating with the AI according to an improved pattern. We asked ChlorTox Analyst to evaluate another eight synthetic procedures from the same course book. In one case, the recipe did not specify the reaction product (it was supposed to be predicted by the student), so we asked the chatbot to make this prediction. The results of this part of the experiment are summarized in **Table** **2**. In this round, the number of identified errors decreased significantly, which emphasizes the importance of the “art” of writing prompts and clearly expressing expectations toward the chatbot. Of note, the structure of the unknown reaction product was predicted correctly. Nevertheless, in some cases errors were still diagnosed—for instance, the command formulated in the textbook was not understood and the chatbot used an intermediate instead of the final product to assess the protocol. Thus, it took one or two additional prompts to obtain the correct Total ChlorTox values for the selected reactions.

**Table 2:** Summary of ChlorTox Analyst validation performed in the Phase III of training on a set of eight synthetic procedures described in an academic textbook.

Synthesis product	Identified issues	Total ChlorTox per 1 g of product [g]
Anthranilic acid	–	3.47
Benzanilide	–	5.19
2,2,2‐Trichloroethanol	–	3.52
Benzilic acid (the rearrangement of dibenzoyl)	The product's formula was not given in the recipe and had to be predicted by AI (additional prompt). The prediction was correct. However, intermediates were taken into ChlorTox calculations instead of the final product (the chatbot did not understand the command in the textbook). Thus, additional prompt was required for correction	4.92
4‐(Acetylamino)benzoic	–	3.52
Benzophenone oxime	–	19.24a)
Cinnamic acid	Some chemicals were omitted by the chatbot. Additional 2 prompts were required to recalculate the correct ChlorTox value	35.82
Benzyl alcohol and benzoic acid	Some chemicals were omitted by the chatbot. Additional 1 prompt was required to recalculate the correct ChlorTox value	3.47

a) The synthesis of 1 g of benzophenone oxime is assessed as the least green, with the estimate risk equivalent to using almost 20 g of chloroform. This result is due to the procedure using large amounts of methanol.

The picture that emerges from these studies is nuanced. On one hand, it can be argued that LLMs trained along the lines we outlined may offer significant savings in terms of the time needed to process complex scientific texts and synthetic procedures and to learn greenness metrics. In addition, the user can interact with the LLM quite flexibly—akin to a human‐to‐human conversation—to alter the assumptions of the query, enter additional data, or force a specific action, e.g., recalculation of the result taking into account additional inputs.

On the other hand, we have seen that even with extensive training by a dedicated human expert, our chatbot still made mistakes and hallucinated some answers—it should be noted that the time to catch these mistakes (and their source) was substantial, to a large degree offsetting the speed of AI‐based assessment. Currently, we do not know (or understand) how the trained model would perform on additional examples. Controlling the accuracy of prediction after addition of every new procedure beats the very purpose of using the AI. Also, in our study, we used very detailed synthetic protocols from an undergraduate course manual—by contrast, protocols published in chemistry journals are often more advanced and also reported in a cursory manner, which may pose additional difficulties for AI interpretation (the reader can test this hypothesis with the most extensively trained version of ChlorTox Analyst available at https://chatgpt.com/g/g‐aX5XsKHki‐chlortox‐analyst‐v‐0‐4).

Of note, our experiences align with a recent case study,^[^ ^59^ ^]^ in which college students used ChatGPT (albeit an earlier, 3.5 version) to propose green modifications to known laboratory procedures and to provide appropriate references from specialized literature. While the students found Chat helpful in writing the introductory parts of an essay, they were much less impressed by technical output (e.g., most outputs were vague, references were almost always wrong, including hallucinated DOI numbers).

### 3.3 Can LLMs Generalize GC Metrics?

Notwithstanding the limitations outlined in the previous section, it is at least conceivable that LLMs will ultimately improve with dedicated training on carefully curated and diverse datasets. In our exercise with ChatGPT, we trained a model based on just one GC metric, but one can imagine much more complex models that use different greenness criteria/metrics. In that case, an interesting question is whether LLMs could reconcile different points of view on greenness and arrive at some consensus metric?

A hypothetical yet, in our opinion, rather improbable scenario is that LLMs fed with enough scientific data, opinion pieces, and popular science texts will ultimately derive by themselves such universally applicable criteria of synthetic greenness. This would require some form of emergent abilities, which have, indeed, been seen in LLMs.^[^ ^61^ ^]^ On the other hand, the models may just learn the prevalent opinion, which, additionally, may fluctuate with time as experts’ viewpoints or governmental regulations change. As a case in point related to GC, the widely heralded EU ban on the use of perfluoroalkyl substances (PFAS) has met with strong opposition from the industry actors,^[^ ^62^ ^]^ and the fate and scope of the regulation are now unclear.^[^ ^63^ ^]^ Of course, there is no ambiguity as to the harmfulness of PFAS, and yet, one of the LLM models (Bing) asked whether “PFAS should be banned” is already giving an ambiguous preamble “This is a complex and controversial question that does not have a simple answer.” This may be because LLMs are trained to avoid controversial conversations—but even if so, how can we trust such politically savvy AI to provide us scientifically sound answers regarding the relative greenness? How could we be sure that these answers are not biased by the abundance of PR data? For instance, would a protocol from some company that is also investing heavily in environmentally oriented PR be scored better than a (objectively) greener protocol from an organization that is not as PR‐active? How could we be sure that the overabundance of promotional data would not outweigh recommendations of experts?

These considerations point to an alternative approach in which LLMs are trained with the help of domain experts. Indeed, it is known that LLMs benefit from the so‐called reinforcement learning from human feedback, which is typically used to fine‐tune these models based on human preferences.^[^ ^64^ ^]^ If multiple experts engaged in conversations with LLMs and “discussed” a large corpus of protocols as well as possible metrics versus experts’ preferences, the model should gradually learn from these conversations. Still, this modality may not be robust against pseudotraining (in the sense that possible counter‐opinions of nonexperts could weigh as much as the expert guidance). This would require developing a fair key for selecting experts for AI learning or differentiating their opinions using weights related to, for instance, experience in GC. Moreover, recent work established that making LLMs larger and more instructible (including utilizing more human feedback) may also make them, perhaps unexpectedly, less reliable.^[^ ^65^ ^]^ In other words, we have no guarantee that more expert training would lead to more robust or generally accepted greenness evaluations.

In addition, one would have no certainty about how and what these LLM really learn, which relates to the important issues of interpretability, trustworthiness,^[^ ^66^ ^]^ and explainability of such “black box” AI systems. With AI recommendations potentially guiding experimental choices or even funding decisions, scientists will be increasingly prone to asking for reasons why or why not the methods they envisioned (or already executed) received a certain greenness assessment. In fact, the right to explain is guaranteed by some legal regulations (e.g., EU GDPR^[^ ^67^ ^]^), and the notion of eXplainable AI (XAI^[^ ^68^ ^]^) is rapidly gaining traction.^[^ ^69^, ^70^ ^]^

### 3.4 Beyond LLMs

**Figure 3:**
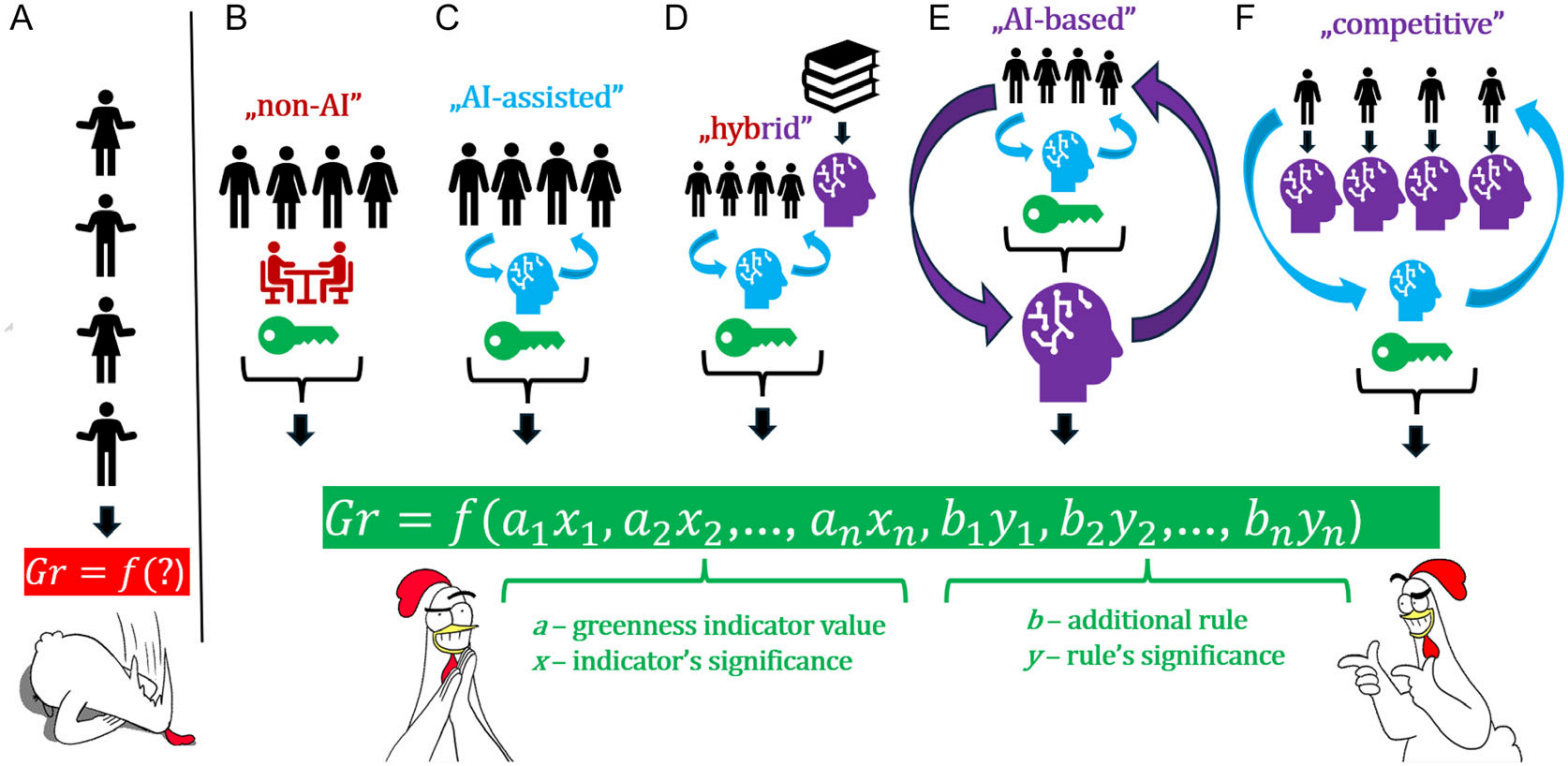
Different possible approaches to the development of universal greenness standards expressed through a mathematical model in which Gr, a hypothetical unified greenness measure, is determined as a function of a number of variables including the results of “*a*” the assessment by different metrics and “*b*” additional rules relating, e.g., to the fulfilment of the particular 12 principles of GC, GAC, and similar assumptions. The weights of these different factors are indicated by “*x* and *y*”. A) Current reality, in which the GC community is fragmented, no universal standards have been developed, and greenness is deemed based on arbitrarily chosen rules. B) A “non‐AI” model, in which standards are developed among GC experts representing a variety of different views, but willing to agree on and jointly establish a compromise. C)—an “AI‐assisted” model with little AI input—limited to helping to find a compromise that satisfies the group of experts; AI does not express its own “opinion” on the form of the Gr function. D) A “hybrid” model, in which AI is treated as “one of the GC experts.” This model is trained based on the available GC literature, at the same time another AI algorithm helps to find a compromise form of the Gr function satisfying the experts. E) An “AI‐based” model, in which the experts do not decide directly on the form of the Gr function, but indirectly, by expressing opinions on AI training guidelines, in the form of the preferred training set components, etc. These experts control the learning process by indicating whether the Gr function proposed by the AI satisfies them or not (it must satisfy the majority of experts); another AI algorithm helps to find a compromise on the training guidelines. F) A “competitive” model, in which experts train many different AIs according to their personal beliefs, which “on their behalf” express the competing Gr function proposals. Another AI algorithm helps to work out a compromise, which is controlled by the experts (it must satisfy the majority of them).

Fortunately, LLMs are not the sole AI modality that may be considered for GC evaluation, and one may envision a more explainable approach based on human expert evaluation of a selected group of chemical procedures, followed by the application of AI/ML to derive a consensus. To illustrate this, let us assume that some number of experts are asked to evaluate the same set of synthetic procedures, {*P*_j_}, each procedure accompanied by a pre‐curated and comprehensive list of variables *x*_*i*_ related to greenness, e.g., atom economy, E‐factor, or outcomes of more complex greenness assessment models. These experts would then be asked to: 1) provide a numeric assessment of each of the procedures (e.g., from 0, not green at all to 100, perfectly green), and 2) mark the variables *x*_*i*_ they feel contributed most to their evaluation. The point of this exercise would be to fit to the distribution of experts’ 0–100 scoring a greenness function, Gr = *f*({*P*_*j*_}, *x*_*i*_) offering the highest correlation with these choices and thus providing a maximally universal metric of greenness. In particular, if Gr were a linear combination of the greenness variables, *x*_*i*_, it could be derived by a standard multivariate regression. In general, assuming that subjective perception and understanding of greenness may partly relate to human psychology, one should probably not expect such linearity. This would imply that the model of Gr be derived via ML techniques. Given the likely limited pool of experts and procedures that could be incentivized to participate in this exercise at the initial stage (say, 20–30 experts and 100–200 procedures), ML would be based on a relatively small set of datapoints, suggesting the use of techniques such as Random Forests, Support Vector Machines, or even simple decision trees. The developed consensus Gr function would keep evolving and, as new data arrives and the group of involved experts expands, the use of more advanced AI methods could be considered. **Figure** **3** illustrates the aforementioned and some more far‐reaching scenarios we envision for the expert‐supervised AI learning.

### 3.5 Some Recommendations on How to Standardize Synthetic Greenness

Regardless of which of these approaches ultimately proves most fruitful, we make three recommendations about structuring this effort: 1) Establish appropriate funding mechanisms. The sheer magnitude of the problem and the need to ultimately ensure community‐wide consensus both dictate that the effort transcends any single research team and, instead, becomes a collaborative, multiteam undertaking. Realistically, this is unlikely to happen without some institutional or governmental incentives—this is always hard to ensure but perhaps not outright impossible given the interest in “green” and “circular” economy the governments prophesize so often. Another source of mecenate could be organizations such as IUPAC (with a mission to foster sustainable development and “provide a common language for chemistry”^[^ ^71^ ^]^) or even LLM companies aiming at robustness (or increased “objectivity”) of their models. Our recommendation is to initiate a granting program(s) aimed specifically at the AI‐driven standardization of synthetic greenness. Our particular suggestion is that such grants should mandate collaboration between parties bringing complementary expertise in synthetic chemistry, process engineering, and ML/AI. Based on our own experience, synthetic chemists are uniquely positioned to analyze the routes for several aspects of the GC 12 guiding principles—for instance, to discern protections and deprotections (especially if more elaborate protection groups are used), to identify stoichiometric chiral auxiliaries versus catalytic reagents, or to determine whether molecules are prone to degradation and, if so, under which conditions. In turn, process chemists or chemical engineers have the requisite skillset to calculate reaction enthalpies, cPMI's or E‐factors, and/or evaluate the syntheses for potential process hazards. The ML/AI codes could be implemented by chemical teams alone (especially if they are based on existing libraries) but, arguably, if algorithmically novel approaches are needed, then computer experts would be of immense help. 2) Establish a ground‐truth dataset of green syntheses. Assuming some granting mechanism becomes available and/or a group of experts nucleates around the problem, the key technical issue will be to amass a body of examples on which the AI is supposed to learn and then test its predictions. The all‐important aspect here is to have some testing examples that serve as “objective truth.” The general chemical literature would likely be of limited value but selecting articles from GC and sustainability‐related journals may provide the first benchmark (vs “random” articles from other sources). More robust benchmarks may also be identified by thorough searching of review articles, source patents, or even company reports. As an illustrative example, in ref. [72], Mark A. Murphy provides a fascinating narrative—and first‐hand knowledge—of the development of industrial‐scale, green, and award‐winning Boots/Hoechst Celanese (“BHC”) process for Ibuprofen's synthesis. This particular article is a treasure trove as it also narrates how the related large‐scale syntheses of acetic acid and its derivatives have evolved over almost a century: from early and wasteful treatments of “pyroligneous liquor” producing stoichiometric amounts of CaSO_4_ wastes, through a greener Celanese's Pampa process using Co salts as catalysts, through the Pd‐catalyzed and water‐based Wacker process, to “Monsanto” Rh‐catalyzed methanol carbonylation and its modern progenies such as Cellanese's Rh‐based “AO” or BP's Ir‐based processes. Data of this kind is particularly attractive because it is accompanied by Celanese's report comparing the environmental differences between various processes.^[^ ^73^ ^]^ One more aspect worth mentioning is that processes at different scales and from different subareas should be considered to provide a balanced representation of greenness metrics (as it is known that, for instance, E‐factors are area‐dependent, varying from around 0.1 in oil refining, to ≈1–5 in bulk chemicals’ production, to ≈5–50 for fine chemicals, and 25–100 for pharmaceuticals^[^ ^74^ ^]^). 3) Establish a public AI–GC repository. Once the datasets are prepared and agreed upon, the infrastructure of the project should be set up in the form of a web portal from which the users could download the datasets, and into which they could deposit their own models and opinions on the solutions proposed by other users. Moreover, to ensure transparency, it would be highly desirable for the users to deposit not only model results but also source codes. While this should not be problematic for ML models developed from scratch by academic groups, there are potential issues with LLMs whose source codes are owned by commercial actors and the best one can hope for is deposition of “conversations” with these models.

## 4 How to Best Popularize AI Models of Synthetic Greenness?

As a final part of this perspective, let us ponder what would be the most efficient way to popularize the AI‐based greenness evaluation schemes we envision? The abovementioned portal aggregating the GC metrics and models (recommendation 3 in the preceding section) would certainly be useful to the groups of developers immediately involved, but may not be frequently visited by practicing synthetic and process chemists who should be the ultimate target audience of the effort. In synthetic chemistry, one typically consults repositories like Reaxys or SciFinder and seeks prior literature precedents and conditions that are not necessarily greener but rather most similar to a reaction of current interest. Indeed, we recently showed^[^ ^75^ ^]^ that conditions chosen to perform reactions tend to repeat those that are already popular in the literature and, in effect, perpetuate the already prevalent trends. Moreover, for certain very complex molecules such as stereodefined natural products, making the product is in itself a major challenge and is often done “at all cost” with consideration of greenness being largely irrelevant.

However, we suggest that these existing biases may be countered by chemical‐synthetic AI itself. In this regard, we observe that recent years have witnessed an explosion of research on algorithms to plan synthetic routes to arbitrary targets autonomously. This almost five‐decade‐old problem, originally pursued by Corey from 1960s onward, has finally been conquered with AI and reaction‐network‐based programs equipped with comprehensive knowledge of chemical transformations.^[^ ^18^, ^19^, ^20^, ^21^, ^22^, ^23^, ^24^, ^25^, ^30^, ^31^ ^]^ Predictions of several of these algorithms have been validated by experiment, including nontrivial targets including drugs^[^ ^19^ ^]^ as well as complex, stereodefined natural products.^[^ ^23^, ^24^, ^76^ ^]^

**Figure 4:**
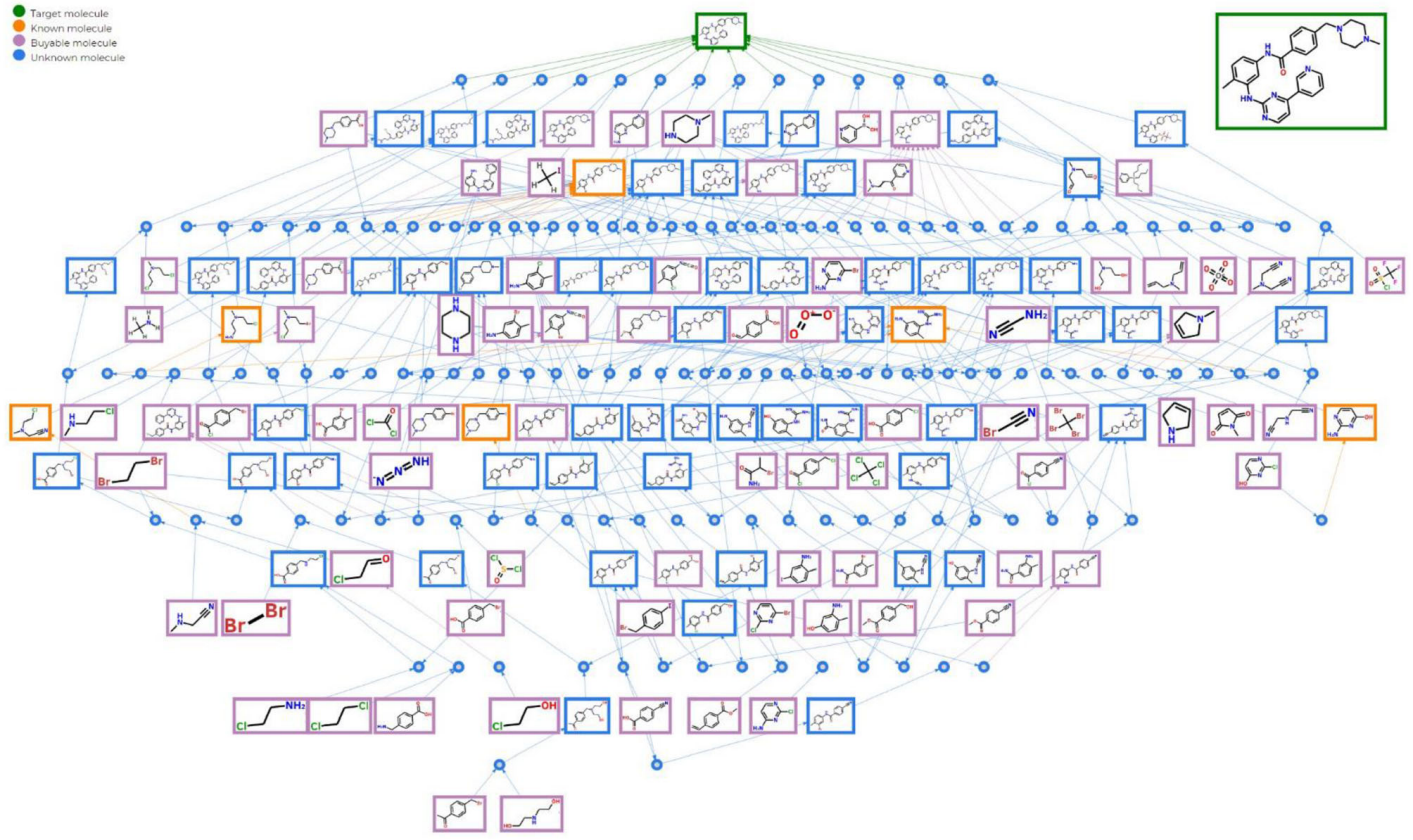
Screenshot of the Allchemy program^[^ ^31^ ^]^ illustrating the multitude of synthetic pathways leading to a simple target, here anticancer imatinib/Glivec drug. Within minutes, the program's retrosynthesis module identifies an entire network of solutions, comprising well over 100 pathways originating from commercially available and inexpensive substrates (chemicals shown in pink boxes). Since the pathways are of comparable lengths and difficulty, it would be desirable to prioritize them not only by process variables (already supported by Allchemy^[^ ^31^ ^]^) but also by their expected greenness.

Important for our discussion is to note that these synthesis‐planning algorithms can often produce tens and sometimes even hundreds of routes to a given target (**Figure** **4**), many of which entail similarly priced substrates, similar convergence, and similar difficulty of synthesis. Traditionally, the routes have been scored and ranked by the economic factors such as the prices of substrates and the cost of synthetic operations.^[^ ^77^, ^78^ ^]^ More recently, however, more attention has been given to other metrics including those of GC. For instance, we have already mentioned in Section 2 that our own Allchemy uses a range of greenness variables (from atom economy, to hazard estimates for various substances and solvents, to PMI indices under different purification schemes) to evaluate the routes, allowing the user to adjust the weights for specific parameters which then enter the overall scoring function.^[^ ^30^ ^]^ Similar schemes have recently been incorporated into Chematica/Synthia^[^ ^79^ ^]^ indicating wider adoption of these concepts. In the future, other metrics of greenness could be incorporated into these scoring schemes (and into other programs such as ASKCOS^[^ ^21^ ^]^ or AiZynthFinder^[^ ^22^ ^]^), including the “universal” metrics we envisioned earlier on.

In parallel, synthesis planning algorithms such as Chematica/Synthia, ASKCOS, Allchemy, or AiZynthFinder rapidly gain wider user base, including in industry. A decade ago, synthesis‐design programs were not used at any of the major chemical or pharma companies. Nowadays, they are deployed in tens (probably close to 100) organizations, and there is no reason to think this trend will not continue, especially that a new, more AI savvy generation of chemists is about to enter the job market.

We feel all these factors play nicely into the GC agenda. One may anticipate that within a decade or so, most synthetic chemists will use one or another synthesis‐design program. If so, it is an immense opportunity for GC to have its metrics incorporated into these programs, such that they become one of the components of the scoring functions, in effect guiding the selection of pathways that are ultimately committed to experiment. This will introduce the green bias into the system from the bottom‐up (i.e., from the programs' users rather than by the force of top‐down regulations), guiding chemists to choose greener solutions they might not otherwise consider (or even be aware of). In other words, we see AI not only as a retroactive tool to perform ex post assessments (to rank the existing protocols), but a proactive component dedicated to an ex ante evaluation integrated with the AI‐based synthesis planning.

## 5 Conclusions

**Figure 5:**
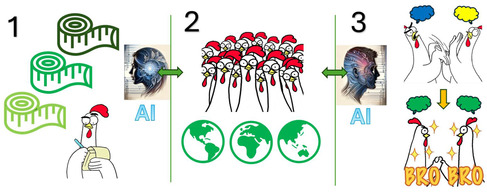
Three intrinsic elements of a comprehensive strategy of giving greenness a more precise meaning: 1) reliable metrics aimed at selected aspects of greenness; 2) a wide group of experts representing different areas of GC, expressing individual opinions; and 3) unification key allowing to find consensus and reconcile opposite views. The potential contribution of AI is expected in points 1 (automated greenness assessment and invention of new metrics) and 3 (greenness unification models). GC experts (point 2) should also control the development and training of AI algorithms and decide on their ultimate application.

In summary, we have argued for an effort in which AI/ML is used to evaluate and potentially also standardize the greenness of synthetic protocols and entire routes. We have shown that already now, state‐of‐the‐art solutions like ChatGPT can analyze scientific texts and evaluate the described synthesis procedures—we see this as encouraging although we also recognize current limitations, such as the propensity to hallucinate some answers and the need for expert supervision (which we consider to be an essential component of any future efforts in this area, see **Figure** **5**). As in any large undertaking, there are significant risks, but there are also realistic remedies (**Table** **3**). One thing we are quite confident about is that GC should try to position itself prominently within the burgeoning area of AI‐based synthesis, to ensure that chemists using these algorithms are guided by comprehensive metrics of greenness.

**Table 3:** Some anticipated difficulties in the development of unified greenness standards and potential remedies.

Potential problem	Preventive measure
Lack of knowledge which methods/algorithms would be optimal	Testing many types of methods in cooperation between the chemical community and IT/AI specialists
Lack of explainability about decisions and assessments (AI models as “black boxes”)	The use of more explainable AI methods (e.g., simple ML as alternative to LLMs) and leveraging the strengths of eXplainable Artificial Intelligence (XAI)
Limited availability and quality of data for training, tendency to replicate the most popular and politically correct opinions	Broad cooperation and involvement of experts controlling the learning process; open discussion of “green standards” in the community)
Lack of expected advances within an assumed timescale, and the significant risk of ultimate project's failure	Increasing the probability of project success through co‐financing and patronage of institutions supporting science

## Supporting Information

Conversations with ChatGPT used as the ChlorTox Analyst.

## Conflict of Interest

B.A.G. is a shareholder of Allchemy company that develops some of the software mentioned in the MS.

## Supporting information

Supplementary Material
